# Liquid state properties and solidification features of the pseudo binary BaS-La_2_S_3_

**DOI:** 10.1038/s41598-021-93576-z

**Published:** 2021-09-14

**Authors:** Charles Boury, Antoine Allanore

**Affiliations:** Department of Materials Science and Engineering, Massachusetts Institute of Technology, Cambridge, MA USA

**Keywords:** Design, synthesis and processing, Phase transitions and critical phenomena, Characterization and analytical techniques

## Abstract

The high temperature thermodynamic properties of chalcogenides materials based on BaS remain elusive. Herein the pseudo binary BaS-La_2_S_3_ is investigated above 1573 K. The liquid properties of BaS-La_2_S_3_ are measured by means of high resolution in-situ visualization coupled with thermal arrest measurements in a thermal imaging furnace. This enables to report the first observation of such melts in a container-less setting. The melting points of BaS and La_2_S_3_ are revisited at 2454 K and 2004 K respectively. La_2_S_3_ demonstrates a high stability in its liquid state, in strike difference with the sublimation observed for BaS. BaS is however partially stabilized with the addition of few percents of La_2_S_3_. The remarkable chemical and thermal stability of La_2_S_3_-rich samples contrasts with the partial decomposition and high vapor pressure observed for BaS-rich samples. Observations and analysis of the solidified samples suggest three different solid solutions. Solid and liquid densities are investigated along the different compositions, supporting a first estimate of the volumetric thermal expansion coefficient for La_2_S_3_.

## Introduction

Sulfides are common compounds on earth and govern society access to a myriad of metals such as copper, nickel, cobalt or even precious metals. As inorganic chemical materials, metal sulfides however have found relatively limited breadth of applications, in part due to their chemical reactivities and the difficulties in their processing. This is in strike difference with the range of properties that they can exhibit, as illustrated by their band-gap which ranges from 1.65 to 3.75 eV^1^. The high temperature behavior of sulfides, in particular those with promising optical properties, remains a frontier that needs to be explored in the context of materials processing. Often, binary or ternary compounds based on mixtures of sulfides are looked for, offering a tunable but unique set of properties. Unfortunately, the most basic high temperature information such as melting point, evaporation rate or range of miscibility are scarce, hindering the development of manufacturing processes.

Barium (+ 2) sulfide (BaS) has been previously investigated for its possible applications in optics, from the stabilization of host glasses^2^ to the generation of luminescent materials when combined with Indium or Gallium sulfides ln_2_S_3_, Ga_2_S_3_^3^. The position of Ba in the periodic table makes BaS the most ionic alkaline rare earth sulfide with a band gap of 2.1 eV^4^ suggesting application in iono-conduction^5^. The standard melting point of BaS has been reported several times at 2508, 2480, 2475, 2470 and 2430 K (respectively^6–10^). Livey *et al.*^9^ mentioned an important volatility for BaS at high temperature impeding the obtention of a stable thermal trace or a good optical visualization. BaS is characterized by a face centered cubic structure (FCC, e.g. NaCl). In contrast with La_2_S_3_, the crystal structure of BaS seems invariant with temperature until its vaporization. The high sensitivity of BaS to air and moisture contamination leads to difficulties in the precise measurements of basic properties such as its melting point as *pure* BaS. In 1968, Bonnivard described the instability of BaS in ambient air^11^. As a white salt when pure, it easily oxidizes at ambient temperature and dissolves in water to a maximum of 500 g per liter. A shift of its color toward yellow is indicative of its oxidation. The overall oxidation products of BaS in ambient air remain uncertain, considering the multiple oxidized compounds possible such as oxysulfides, sulfates, oxides, carbonates....

Stinn *et al.*^6^ investigated the pseudo binary of BaS combined with dicopper (+2) sulfide (Cu_2_S) using a combination of differential thermal analysis and in-situ thermal imaging furnace. This study indicates a large depression of the melting point of BaS by the addition of as little as 5 mol% Cu_2_S. A broad liquid miscibility gap is found, the two-liquids being optically distinct: the BaS-rich liquid exhibits optical features of an ionic melt while the Cu_2_S-rich melt displays optical features closer to Cu_2_S. The results demonstrate the ability to stabilize a melt with up to 95 mol% BaS, which supported a new electrochemical processing technique using BaS-based sulfides electrolytes with un-precedent solubility and electrical conductivity. Cu_2_S is a solid and liquid semiconductor, while BaS is an insulating, ionic compound. It is therefore of interest to evaluate if the electronic nature of the second compound added to BaS affects the melting behavior. This requires to investigate a pseudo-binary system with an ionic second compound, and lanthanum (+3) sesquisulfide (La_2_S_3_) is a good candidate.

Unfortunately, knowledge about La_2_S_3_ is also scarce. Flahaut *et al.* discovered the remarkable homogeneity of rare-earth ceric sulfides (ceric elements: La, Ce, Pr, Nd, Pm, Sm, Eu)^12–17^ demonstrating commonalities in crystal structures, melting points and oxidation. Andreev *et al.* experimentally explored the pseudo-binary phase diagrams of metallic (+2) sulfides - rare earth (+3) sulfides, describing similarities and differences in properties^18–25^.

For La_2_S_3_ and other ceric (+ 3) sesquisulfides, different solid phases, $\alpha$, $\beta$, $\gamma$, have been reported^26^. $\alpha$-La_2_S_3_, with an orthorhombic structure, is stable until 1173 K. $\beta$-La_2_S_3_ has a stability domain from 1173 K to 1573 K and exhibits a tetragonal structure, similar to the oxysulfide ${\text {La}}_{10}{\text {S}}_{14+x}{\text {O}}_{1-x}$. The high-temperature stable phase, $\gamma$-La_2_S_3_ has a cubic ${\text {Th}}_3 {\text {P}}_4$-type structure, with composition ${\text {La}}_{3-x}{\text {V}}_x{\text {S}}_4$ (0 $\le$ x $\le$ 1/3, ranging between ${\text {La}}_3{\text {S}}_4$ and La_2_S_3_), where V represents La vacancies. The $\beta$ and $\gamma$ phases are unstable at room temperature, however $\gamma$-La_2_S_3_ can be stabilized by introducing alkali ions^27^ or europium^28^. Furthermore Kumta *et al.* found a process to generate and stabilize fine $\beta$-La_2_S_3_ and $\gamma$-La_2_S_3_ powder at high temperature^29^. Two other phases $\delta$ (monoclinic) and $\epsilon$ (rhombohedral) exist for some sesquisulfides but have not been reported for La_2_S_3_^1^. Prior studies of La_2_S_3_ intended to determine its chemical stability with respect to oxygen and sulfur as a function of temperature. In 1981, Kamarzin *et al.* studied lanthanum (+3) sesquisulfides from LaS to La_2_S_3_^30^. In 1984, Kay *et al.* proposed a phase stability diagram for lanthanum versus the vapor pressure of ${\text {O}}_2$ and ${\text {S}}_2$ at 1100 K^31^. Vasilyeva described the thermodynamic of the La_2_S_3_-${\text {LaS}}_2$ system in 2010^32^.

The melting point of La_2_S_3_ has been investigated several times^10,15,30,33,34^, reported between 2133 and 2350 K. The potential reactivity with crucibles might have affected the accuracy of the results. In addition, the vaporization of La_2_S_3_ before its melting has been reported at around 2000 K. Flahaut and Picon investigated the lanthanum oxide (${\text {La}}_2{\text {O}}_3$) and oxysulfides (${\text {La}}_2{\text {O}}_2$S) and concluded their formation from sulfide was kinetically slow^35,36^. Sulfates ${\text {La}}_2 ({\text {SO}}_4)_3$ and oxysulfates ${\text {(LaO)}}_2$ ${\text {SO}}_4$ require specific conditions to be formed from sulfides, unattained herein^37,38^.

La_2_S_3_ has found applications in certain glasses, $\beta$-La_2_S_3_ potentially exhibits properties of phosphorous material^39^. La_2_S_3_ also found usage as an electrode in its $\alpha$ phase for its pseudo-capacitive behavior when immersed into a ${\text {Na}}_2$ ${\text {SO}}_4$ electrolyte^40,41^. From the common crystal structure between La_2_S_3_ and ${\text {La}}_3{\text {S}}_4$ at low temperature ($\alpha$ phase), superconducting properties has been observed with a Curie point varying with the metal vacancy concentration^42^. The transformation of La_2_S_3_ to ${\text {La}}_3{\text {S}}_4$ leads to drastic changes in the sulfide electrical properties; Wood *et al.* reported an insulator behavior of La_2_S_3_, compared to ${\text {La}}_3{\text {S}}_4$ which acts as a semi-metal^43^.

The combination BaS-La_2_S_3_, despite very limited prior art, supported the extension of molten sulfides electrolyte based on BaS, in particular for copper electrowinning from ${\text {Cu}}_2$S^44^. BaS-La_2_S_3_ was postulated to bring two essential properties for the electrolysis of ${\text {Cu}}_2$S into ${\text {Cu}}_{(l)}$ and ${\text {S}}_{2(g)}$: the wide band-gap of BaS allows ionic conduction and the addition of La_2_S_3_ decreases the melting point of the electrolyte.

Herein, the liquid state properties and solid phases found upon solidification of the pseudo binary BaS-La_2_S_3_ are investigated. The use of a container-less thermal imaging furnace allows to observe unique features such as molten state stability, melt density and evaporation processes. The liquidus line and melting points of BaS and La_2_S_3_ are reported and compared to the literature. Mass loss, porosity and liquid state behavior are investigated to highlight the differences between BaS-rich samples and La_2_S_3_-rich samples. Following solidification, different solid solutions are observed and compared to the literature, allowing to propose a preliminary version of the pseudo binary BaS-La_2_S_3_ phase diagram. Some hypothesis regarding the oxidation processes of alkaline sulfides (BaS) and rare-earth sulfides (La_2_S_3_) are proposed to compare the analyzed phases with the literature. Preliminary results for densities of molten and solidified samples are reported. In closing, an attempt to determine the volumetric isobaric thermal expansion coefficient of La_2_S_3_ is presented.

## Experimental and analysis

### Samples preparation

The melting behavior is investigated along the pseudo-binary system ${\text {(BaS)}}_x$-$({\text {La}}_2 {\text {S}}_3)_{1-x}$, with increments *x* of 10 mol%. The isothermal melt stability over long durations is studied for several temperatures for two specific compositions: ${\text {(BaS)}}_{0.25}$-$({\text {La}}_2 {\text {S}}_3)_{0.75}$ and ${\text {(BaS)}}_{0.75}$-$({\text {La}}_2 {\text {S}}_3)_{0.25}$. Four samples of both compositions are set at different temperatures (2133, 2023, 1896, 1889 K and 2233, 2160, 2129, 2093 K respectively) to investigate the melt long term stability.

BaS and La_2_S_3_ powders come from Alfa Aesar with a respective purity of 99.97 % and 99.99 % (metal basis). Each sample is prepared in a glove box with atmosphere control using argon, indicating a maximum oxygen content of 10 ppm. Each powder is weighed, mixed and milled into a mortar and pestle. The homogeneous mix is poured into a cleaned rubber balloon. The balloon is stretched and compressed to obtain a rod-like shape of homogeneous density and diameter, then a knot is tight at both ends. With both ends attached to a stainless steel support to maintain a rod-like shape, the balloon is placed into an hydrostatic press and compressed to 30000 psi (2068 bar). After compression, the rod is removed with care by cutting along the rubber balloon with a stainless-steel scalpel in a fume hood. The rod is maintained at the end of a moveable and rotating central shaft of a thermal imaging furnace by means of a tungsten or molybdenum wire. The time required to assemble the rod and set it up in the furnace involves contact with ambient atmosphere for around 1 hour. The compacted rods are slightly friable prior to melting, influencing to a certain extend the repeatability of mass loss measurements.

### Thermal imaging furnace

**Figure 1:**
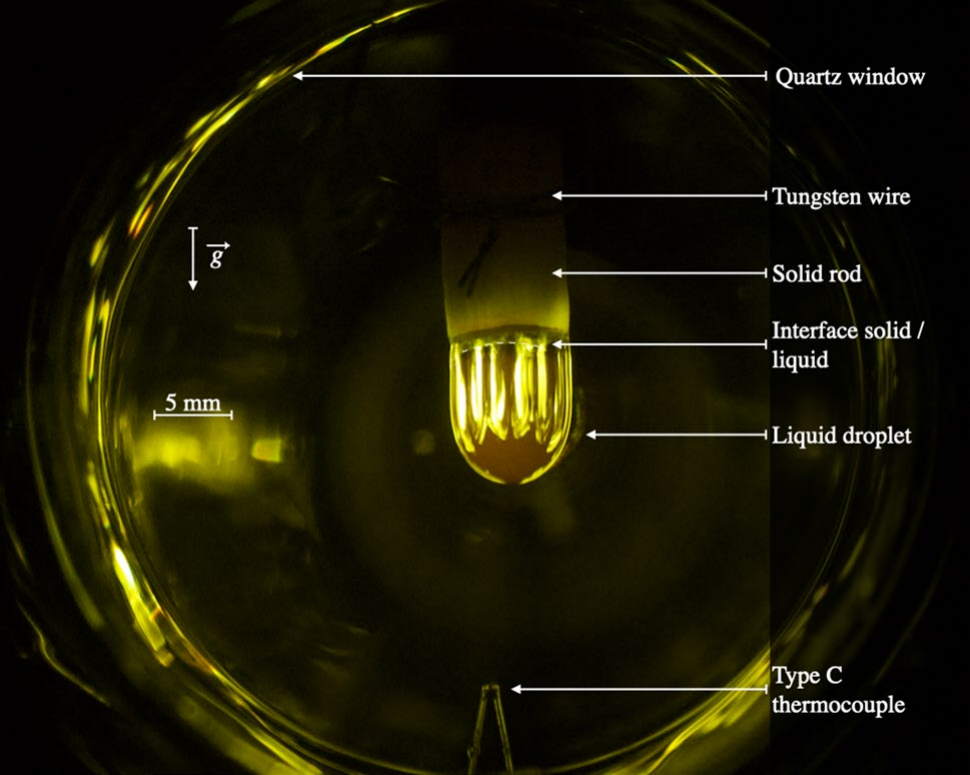
Picture of a liquid La_2_S_3_ droplet pending from its solid formed in the Thermal Imaging Furnace. (Camera Canon Inc., EOS Rebel T5i DSLR, EF-S 18–135 mm).

Both BaS and La_2_S_3_ are compounds with melting temperature above 1900 K, requiring special attention with respect to materials compatibility, few refractory materials being compatible. Instead herein a container-less configuration is used in a thermal imaging furnace (Crystal Systems Corp., model TX-12000-I-MIT-PC). It is equipped with four Xenon lamps of 3 kW of power each. The lamps irradiate a center cone of light of around $1 \, {\hbox {cm}}^3$, in which the lower end of the sample is placed by moving the central shaft down. There, the sample is heated up by light/matter interaction. Temperatures greater than 3300 K have been obtained on a tungsten sample. A view of a liquid sample in place (La_2_S_3_) is shown in Fig. 1. Compared to traditional furnaces based on resistance heating, high temperatures can be reached «instantaneously »avoiding long heating processes and as evidenced from Fig. 1 observation and mechanical access to a droplet is relatively straightforward.

**Figure 2:**
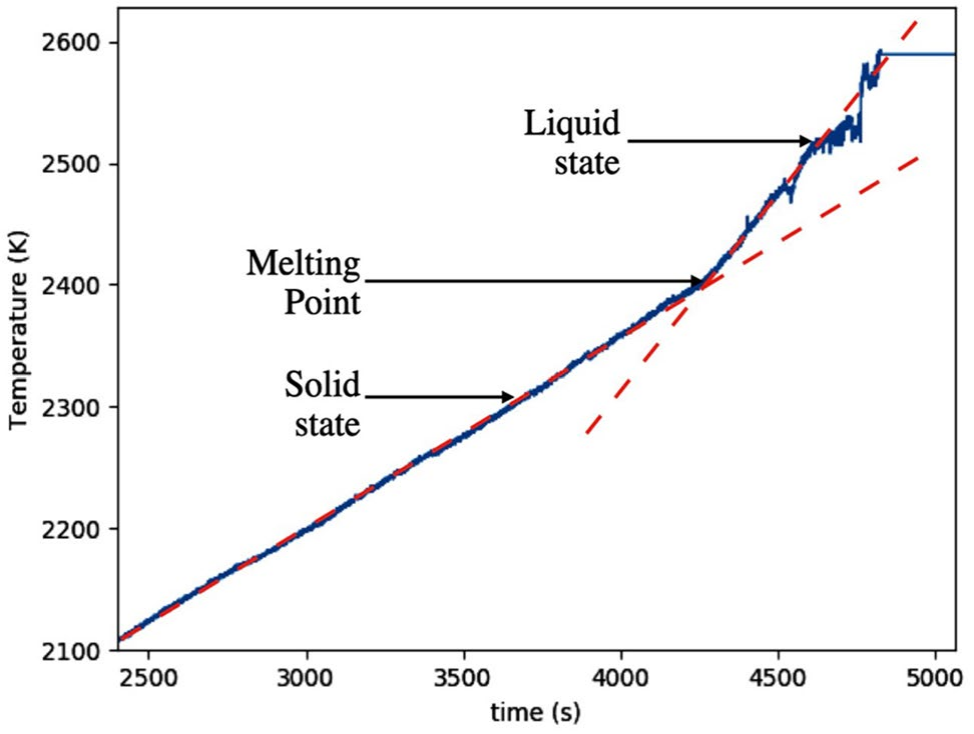
Example of the thermal trace obtained from the thermocouple located into BaS sample. The two dotted lines are used to evaluate the melting point.

Visual access to the samples is possible between the lamps on the 4 sides of the thermal imaging furnace. A camera (Watec, WAT-233 1/3”, Extender EX2C, Computar Co., Fujinon TF4XA-1) equipped with a UV filter is fixed to the front door and used to observe samples live. A second camera (Canon Inc., EOS Rebel T5i DSLR) equipped with a zoom lens (Canon Inc., EF-S 18–135 mm) is set up on a tripod orthogonal to the front door. Melting and solidification processes are observable, either in live mode or recorded. A feed-through at the bottom of the quartz tube allows to insert a type C thermocouple (W-Re 5%, W-Re 26%) for direct temperature measurement of the melt. The thermocouple signal is converted into temperature following the Reference Tables N.I.S.T. Monograph 175- Revised to ITS-90. The reference junction temperature is corrected by 17 K to consider the actual room temperature. The thermocouple signal limits of error is 1 % of the read value in the range 273 K to 2600 K. A frequency acquisition of 3 Hz is typically used, increased to 1000 Hz when monitoring rapid cooling. Simple thermometry^45^ is used to study the liquidus temperature as illustrated in Fig. 2. Other methods were not found yet compatible with the thermal imagining furnace. For a given sample, the liquidus temperature is crossed several times from visual observation, and several traces are then used to provide an average value.

### Method for ${\text {(BaS)}}_x$-La_2_S_3_

When the furnace is turned on at the minimum attainable power (0 % power), the Xenon lamps are already emitting light leading to a minimum temperature of around 1550 K to 1650 K for the samples investigated herein. This value is dependent on the specific sample materials, its geometry, and the furnace configuration. Herein, 1% of power increment leads to 30 to 40 K increase when the sample is solid. A first slow increase at around 1 % power min^−1^ is used to reach the melting point and not overheat the sample. When interested in the solid-phases, the power is decreased gradually to 0 % power and samples solidify up to $400 \, {\hbox {K.s}}^{-1}$ when the furnace is turned off. For the investigation of the liquidus, the ramp rates range from 0.2 to 4% min^−1^. The melting point or liquidus temperature are measured by repeating heating and cooling processes across the phase transition until the thermal trace shows a reproducible profile.

For all samples, a gaseous volume is formed inside the liquid droplet during a first melting of a sample. This gaseous volume has several origins, from the initial porosity consequence of the rod processing, to the chemical generation of a sulfuric gaseous phase composed of elemental sulfur S and including traces of metallic elements. The sulfuric gas phase has a yellowish color and is mainly observed for BaS-rich samples. Primary vacuum is applied several times during each experiments to extract this gas phase and ensure a fully liquid composition of the droplet for liquid density measurements. In the case of BaS-rich samples, the formation of a neck is observed at the solid/liquid interface when the sample stay in its liquid state for more than half an hour. The formed neck can be re-melted by slightly lowering the sample into the hot zone, for example for liquid density measurements. The droplet is considered deprived of gaseous phase when no visible effect is observable during the application of primary vacuum.

### Density measurements

Pictures from camera recordings along with pixel size determination are used to calculate the equivalent volume of liquid, based on the method detailed by Wu *et al.*^46^. Four pictures per sample are analyzed to average the volume and minimize the possible departure from axi-symmetry. After the experiment, the solidified droplet is separated from the rest of the sample with a stainless-steel scalpel, and weighed. The distinction between the melted and non-melted part is clear on the La_2_S_3_-rich samples. The neck formation with the BaS-rich samples makes this distinction more tedious. The weighted mass of solidified droplet and the liquid volume from the pictures provide an estimate of the liquid density. Archimedes’ law is used to evaluate the density of the solidified droplet (specific gravity kit from Mineralab, using air and ethanol).

### Elemental analysis

This work does not have as primary objective the determination of the solid-state phases for the pseudo-binary phase diagram. However, important observations on the elemental composition of the solidified phases are reported. Droplet samples are cast in epoxy and grounded along to gravity with silicon carbide paper (Grit 4000) using ethanol as a lubricant. BaS and La_2_S_3_ samples were polished down to 1 micrometer. Elemental analysis for those samples is conducted with a scanning electron microscope equipped with Wavelength Dispersive Spectroscopy microprobes (WDS, JEOL JXA-8200 Super-probe). Other samples were observed and analyzed on a scanning electron microscope (SEM, JEOL JSM- 6610LV, JEOL Ltd.) equipped with a single Energy Dispersive Spectroscopy detector (EDS, Sirius SD detector, SGX Sensor-tech Ltd.). Several factors are important when considering elemental analysis of the solids phases as reported herein:

1. Solidified BaS-La_2_S_3_ samples have a low electronic conductivity and despite the use of conductive tapes, high definition images proved difficult to obtain at a greater than 20,000x.
2. Ba and La have characteristics X-rays close to each other (L$\alpha$: 4.465 keV, 4.650 keV and M: 0.972 keV, 0.833 keV respectively).

### Oxidation

BaS-La_2_S_3_ samples are unstable in air, leading to potential oxidation after exposure to atmosphere, and some oxygen content was found with EDS or WDS. As found from the literature, oxygen contamination of lanthanum sulfides is mainly due to the formation of ${\text {La}}_2{\text {O}}_3$ or ${\text {La}}_2{\text {O}}_2$S while the oxidation of BaS mostly lead to BaO. Without known mixed Ba-La oxide or oxysulfides, the elemental ratio Ba / La is considered constant and independent of the oxidation state so that elemental analysis results are reported as the ratio ([Ba]/([Ba]+[La])). WDS, contrary to EDS, can provide quantitative elementary analysis of S and O, and oxidation is assumed to a lead to a stoichiometric substitution of S by O. The elemental concentration of S + O is then equal to the concentration of S from the as-solidified sample recovered from the furnace.

A small quantity of powderous material from condensation on the quartz tubes is recovered and analyzed. Its difficult acquisition leads to high uncertainty in its composition. The presence or absence of metallic compound can however be discussed.

## Results

### Lanthanum (+3) sulfide and barium (+2) sulfide

Pure La_2_S_3_ generates a small volume of visible gas during the first heating process. A mass loss of 1.5 wt% is observed. S, and La in a minor extent are detected with EDS on the condensate recovered on the quartz tube. After this first melting, La_2_S_3_ demonstrates a stunning stability as a liquid phase, never reported before in the literature. Pure BaS generates a non-quantifiable volume of visible gas both as solid upon heating or as liquid. BaS decomposes before melting, producing barium and sulfur vapors that condensate on the quartz tube. No metallic barium is found inside the sample droplet, indicative of a vaporization of both Ba and S, in agreement with the composition of the condensates. As described in the literature, ${\text {BaS}}_{(l)}$ cannot be stabilized under argon at atmospheric pressure.

**Figure 3:**
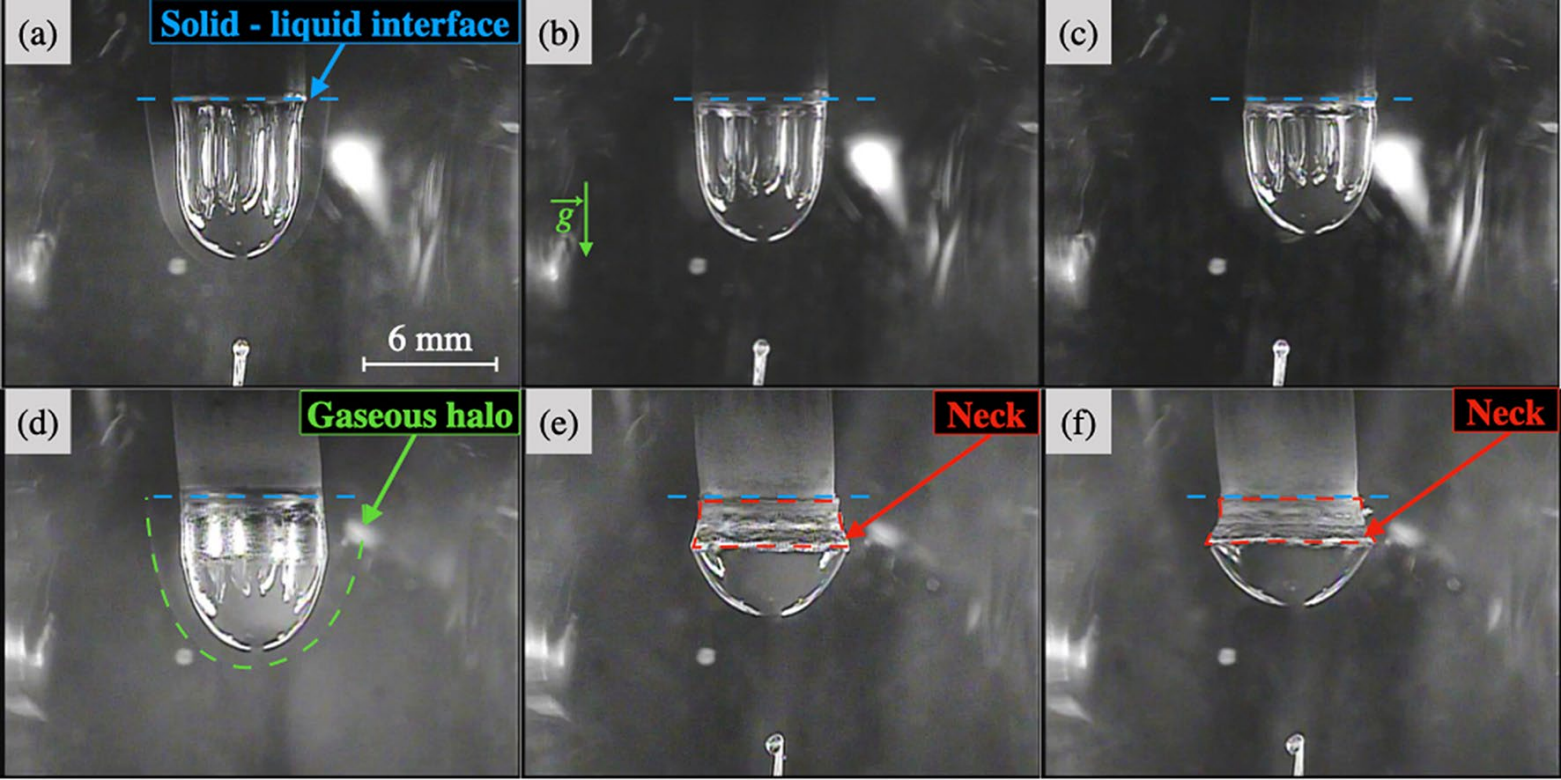
Study of the variation of the shape of the droplets during an experiment at constant temperature. Pictures of ${\text {(BaS)}}_{0.25}$-$({\text {La}}_2 {\text {S}}_3)_{0.75}$ droplet shape (**a**) at the beginning, (**b**) during, (**c**) at the end of the experiment; ${\text {(BaS)}}_{0.75}$-$({\text {La}}_2 {\text {S}}_3)_{0.25}$ droplet shape (**d**) at the beginning, (**e**) during, (**f**) at the end of the experiment. (Camera Watec equipped with a UV filter).

**Figure 4:**
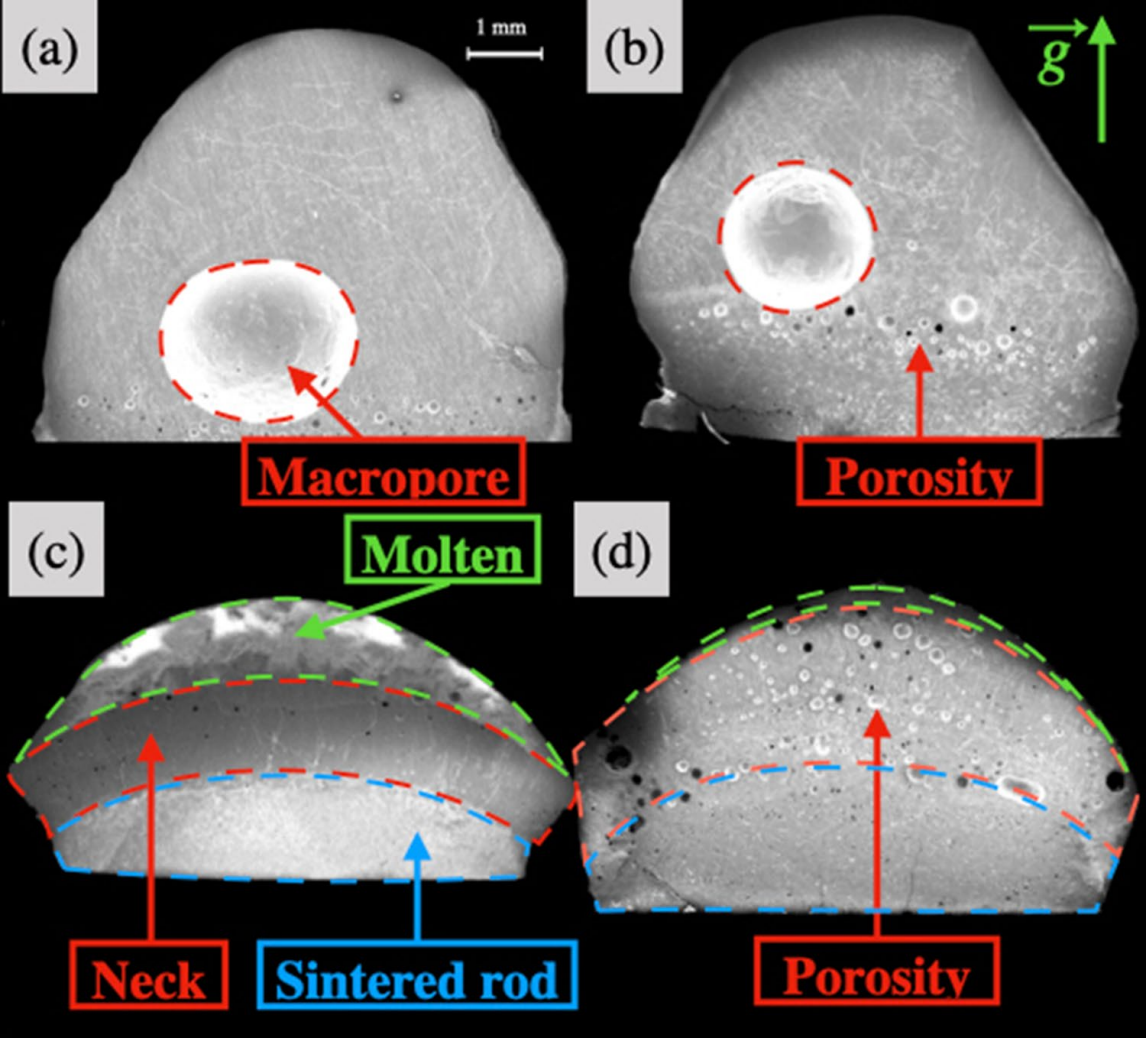
SEM (JEOL JSM- 6610LV, JEOL Ltd.) images of ${\text {(BaS)}}_{0.25}$-$({\text {La}}_2 {\text {S}}_3)_{0.75}$ solidified droplets maintained at (**a**) 2023 K and (**b**) 1896 K; ${\text {(BaS)}}_{0.75}$-$({\text {La}}_2 {\text {S}}_3)_{0.25}$ droplets maintained at (**c**) 2233 K and (**d**) 2129 K.

### La_2_S_3_-rich samples

La_2_S_3_-rich samples exhibit thermal features that lead to prompt homogenization of the temperature inside the droplet. Analogous melting behavior is found for 50 to 90 mol% La_2_S_3_, and results for ${\text {(BaS)}}_{0.25}$-$({\text {La}}_2 {\text {S}}_3)_{0.75}$ are specifically presented. Views of the droplet at 3 stages during a 45 min experiment are found in Fig. 3a–c. The gaseous halo is of mild intensity and observed for a short amount of time (from a minute at 1889K to twenty minutes for 2133 K). The mass loss is low, and moderately sensitive to temperature, with 3 % at 1903 K and 4 % at 2123 K. Traces of Ba and La have been collected on the quartz tube for all four experiments. The shape of the stabilized liquid droplets, as shown in Fig. 3a–c is very similar to that of pure La_2_S_3_ shown in Fig. 1.

Figure 4a,b shows the secondary electron images of the cross section of the solidified droplet observed in the SEM. Macroscopically, a single phase as well as a macropore of few millimeters are observed for all La_2_S_3_-rich compositions, regardless of the temperature. This macropore is not found on solidified samples if the droplet is exposed to several cycles of controlled vacuum/atmospheric melting. The small porosity observed at the bottom of the pictures is always present and attributed to the initial solid-rod porosity.

### BaS-rich samples

BaS-rich samples do not exhibit favorable thermal properties and are difficult to melt. A thermal gradient is present and it proved possible to observe a liquid surface while the thermocouple remains mechanically entrapped in the core solid. BaS-rich liquid behavior is complex and cannot be described only through the analysis of ${\text {(BaS)}}_{0.75}$-$({\text {La}}_2 {\text {S}}_3)_{0.25}$ samples, representing long term stability liquid behavior. The mass loss during ${\text {(BaS)}}_{0.75}$-$({\text {La}}_2 {\text {S}}_3)_{0.25}$ experiments reaches 10 % for the lowest temperature (2103 K) and increases to 15 % at higher temperature (2233 K). Mass loss observed with other experiments on BaS-rich samples is not representative, droplets often fell or exploded. A larger quantity of powder is deposited on the quartz tube for BaS-rich samples than La_2_S_3_-rich samples. Ba has been rejected on the quartz tube, no La has been found.

Figure 3d–f represent the shape of the molten droplet ${\text {(BaS)}}_{0.75}$-$({\text {La}}_2 {\text {S}}_3)_{0.25}$ at three different times of the experiment. The neck forms slowly along with a diminution of the volume of liquid compared to its initial volume. For a constant lamp power, the neck melts instantaneously when the upper part of the sample is lowered into the hot zone. Figure 4c–d are SEM/EDS images of the ${\text {(BaS)}}_{0.75}$-$({\text {La}}_2 {\text {S}}_3)_{0.25}$ samples at 2233 K and 2129 K, illustrating the final flattened shape, the neck position and the presence of multiple macrophases. The neck forms at the initial frontier of the solid and the liquid.

For ${\text {(BaS)}}_{0.75}$-$({\text {La}}_2 {\text {S}}_3)_{0.25}$ long term experiment, as show on Fig. 4c–d, a porosity consisting on a sporadic repartition of pores with diameters of one-tens of a millimeter is observable. Increasing the temperature enhances the volume of visible gas generated. If no vacuum is applied during a long experiment, the porosity seems to decrease with the increase of temperature or time spent in the liquid state. However when BaS-rich samples are melted for few minutes and the neck formation is at its initial stage, the fast solidification without the application of vacuum leads to a macropore similar to the one observed for La_2_S_3_-rich samples. This macropore is not found with repeated application of vacuum.

**Figure 5:**
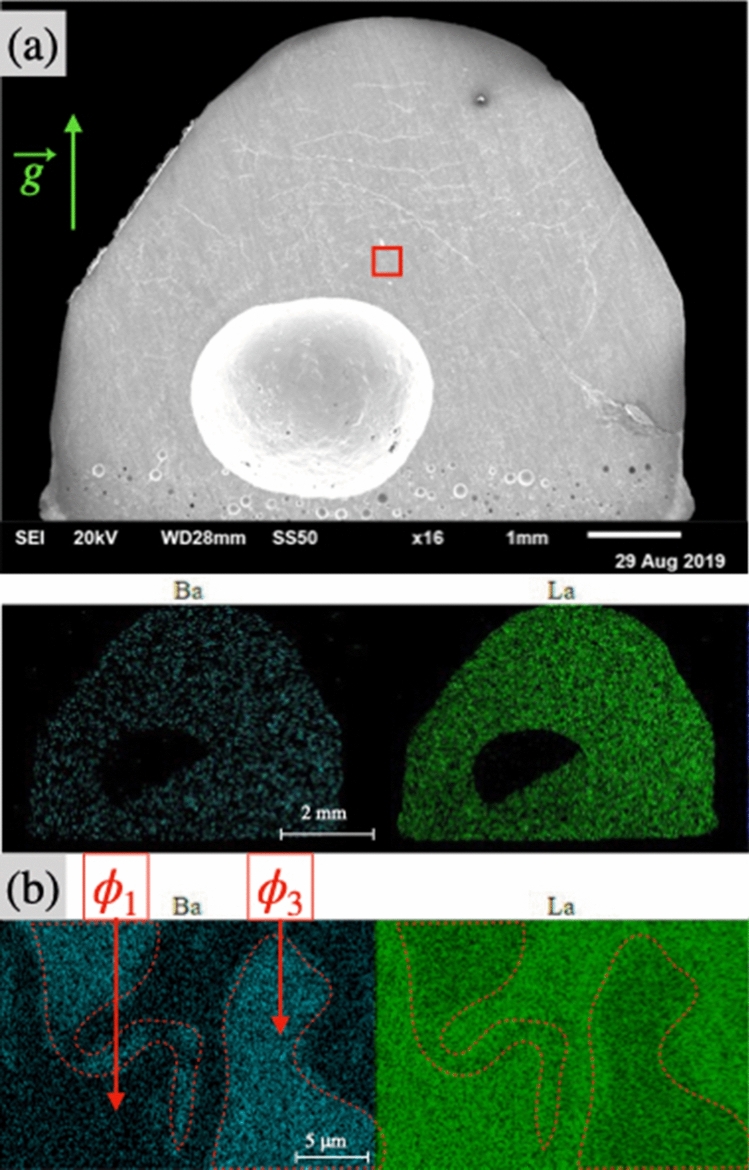
(**a**) SEM equipped with a single EDS detector images of ${\text {(BaS)}}_{0.25}$-$({\text {La}}_2 {\text {S}}_3)_{0.75}$ sample acknowledging a single macrophase; (**b**) zoom on the red rectangle to observe the $\phi _1$ and $\phi _3$ phase separation on a microscale for ${\text {(BaS)}}_{0.25}$-$({\text {La}}_2 {\text {S}}_3)_{0.75}$ sample.

**Figure 6:**
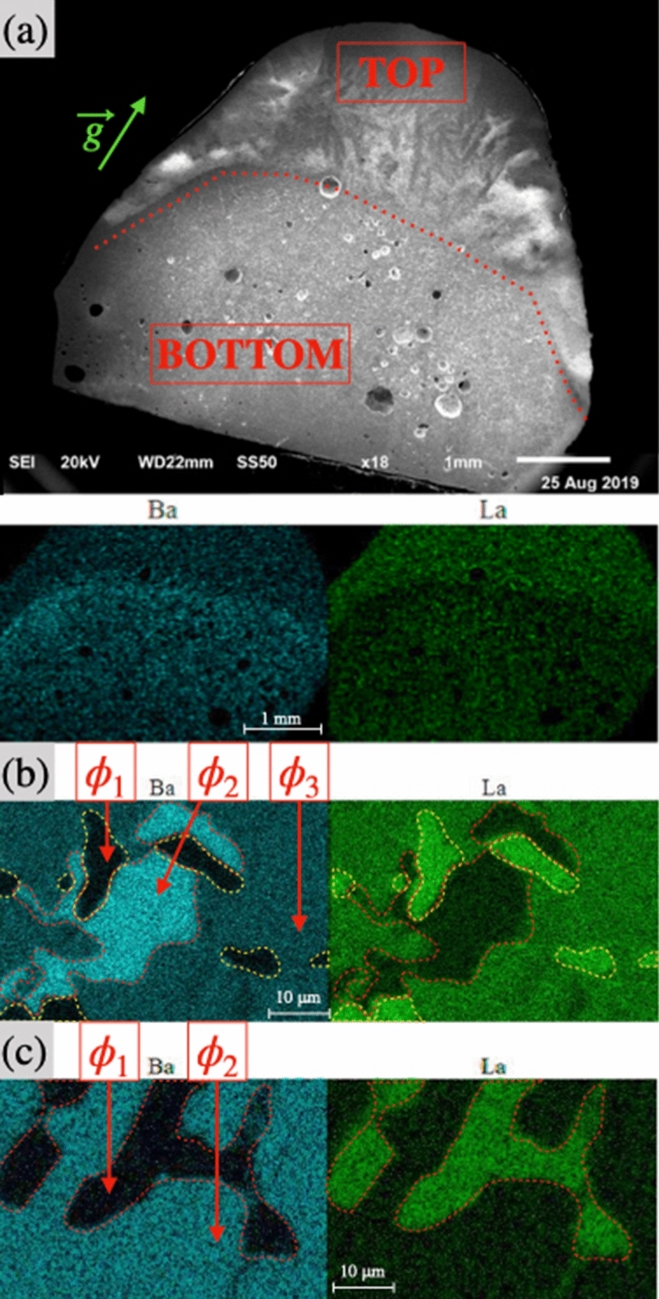
(**a**) SEM equipped with a single EDS detector images of ${\text {(BaS)}}_{0.30}$-$({\text {La}}_2 {\text {S}}_3)_{0.70}$ sample acknowledging different macrophases; (**b**) example of $\phi _1$, $\phi _2$ and $\phi _3$ phase separation for ${\text {(BaS)}}_{0.25}$-$({\text {La}}_2 {\text {S}}_3)_{0.75}$ samples; (**c**) example of $\phi _1$,$\phi _2$ phase separation for ${\text {(BaS)}}_{0.30}$-$({\text {La}}_2 {\text {S}}_3)_{0.70}$ samples.

### Composition of the solidified droplets

Three solid phases, named $\phi _1$, $\phi _2$ and $\phi _3$ are observed, distinguished by their relative barium content presented in Table 1. $\phi _1$ is characterized by a maximum ratio of barium to the other metallic elements of 9 %. In $\phi _2$ this ratio is at minimum 90 %. $\phi _3$ is characterized by a ratio between 23 to 42 %. ${\text {La}}_2 {\text {S}}_3$-rich samples are characterized by a single macrophase, as represented on Fig. 5a. The sample containing 50 mol% ${\text {La}}_2 {\text {S}}_3$ is mostly the single homogeneous $\phi _3$ solid solution, with traces of $\phi _1$ and $\phi _2$ present throughout the sample. Unfortunately, ${\text {(BaS)}}_{0.4}$-$({\text {La}}_2 {\text {S}}_3)_{0.6}$ sample could not be characterized. Samples containing from 70 to 80 mol% ${\text {La}}_2 {\text {S}}_3$ are composed of two phases, $\phi _1$ and $\phi _3$ with grain size of few tens of micrometers as shown on Fig. 5b. Sample containing 90 mol% of ${\text {La}}_2 {\text {S}}_3$ is mainly composed of $\phi _1$, with some traces of $\phi _3$. The ratio of barium to metallic elements increases in $\phi _1$ and $\phi _3$ with the increase of the initial BaS concentration. The duration of the experiment and the operating temperature does not seem to affect the composition of the different solid solutions observed.

**Table 1:** Phases observed over the BaS-${\text {La}}_2 {\text {S}}_3$ pseudo-binary phase diagram.

Phase $\phi _i$	[Ba]/([Ba]+[La])
$\phi _1$	0 to 9%
$\phi _2$	90 to 100%
$\phi _3$	23 to 42%

For BaS-rich samples, different macrophases are observed as shown on Figs. 4c,d and 6a. The presence of $\phi _1$, $\phi _2$, and $\phi _3$ solid solutions have been observed in the BaS-rich samples and their presence are highly dependent of the parameters of the experiment (i.e. duration, cooling rate, remelt of the neck). For long term stability experiments conducted with ${\text {(BaS)}}_{0.75}$-$({\text {La}}_2 {\text {S}}_3)_{0.25}$ samples (Fig. 4c,d), the neck region is in majority $\phi _2$ with some $\phi _3$, whereas the molten region is mostly $\phi _3$ solid solution with some $\phi _2$. Traces of $\phi _1$ are observable mostly in the sintered region as seen in Fig. 6b.

Between 10 and 40 mol ${\text {La}}_2 {\text {S}}_3$, the sample is lowered by few millimeters in the hot zone to melt the neck and primary vacuum is applied several times in order to take liquid density measurements. BaS-rich samples exhibit a majority of $\phi _2$, and $\phi _3$ solid solutions. However at 30 and 40 % ${\text {La}}_2 {\text {S}}_3$, $\phi _1$ and $\phi _2$ are largely observed while the presence of $\phi _3$ solid solution tends to decrease. As shown in Fig. 6c the solidification of ${\text {(BaS)}}_{0.70}$-$({\text {La}}_2 {\text {S}}_3)_{0.30}$ sample led to a phase separation in $\phi _1$ and $\phi _2$ solid solutions.

### WDS results

The average composition over 20 WDS points for the lanthanum sulfide leads to ${\text {La}}_{0.394}{\text {S}}_{0.606}$, close to the theoretical ${\text {La}}_{0.4}{\text {S}}_{0.6}$. The standard deviation for La is 0.445 mol% and the sum of the standard deviations of S and O reaches 2.167 mol%. The oxidation of ${\text {La}}_2 {\text {S}}_3$ seems slow, with only few percent of oxygen found. A single point showed a high concentration of oxygen, likely representing a fully oxidized inclusion.

The results are more scattered for BaS, with an atomic concentration between 37 to 51 mol% Ba, 2 to 30 mol% for O, and 31 to 47 mol% for S. The formation of different barium oxides, oxysulfides and oxysulfates support this range of variation. Only the results with a low percentage of oxygen are further considered. The average experimental composition is then ,${\text {Ba}}_{0.506}{\text {S}}_{0.493}$, quite close to BaS. The standard deviation for Ba is 1.237 mol% and the sum of the standard deviations of S and O reaches 1.297 mol%.

### Liquidus (Table 2)

Table 2 represents the liquidus temperature measured for the BaS-${\text {La}}_2 {\text {S}}_3$ pseudo-binary compounds. The mean value for the melting point of BaS is 2454 K and 2004 K for ${\text {La}}_2 {\text {S}}_3$. The addition of few percent of ${\text {La}}_2 {\text {S}}_3$ into BaS leads to an important decrease of the liquidus temperature. The minimal melting temperatures are around 25 mol% and between 86 mol% ${\text {La}}_2 {\text {S}}_3$, representative of two eutectic behaviors. For ${\text {La}}_2 {\text {S}}_3$-rich samples, the gas generation is visibly limited, and the final elemental composition is close to the initial one. However, for BaS-rich samples, the important gas generation and sensibility to ambient air may lead to a slight shift of the initial BaS composition. Liquidus measurements are conducted as promptly as possible to stay close to the initial composition.

**Table 2:** Liquidus temperature (K) and densities (${\text {g cm}}^{-3}$) for the initial concentration of ${\text {La}}_2 {\text {S}}_3$ (mol%), «- »values not accessible.

mol% ${\text {La}}_2 {\text {S}}_3$	0	10	20	30	40	50	60	70	80	90	100
Temperature (K)	2454	2149	1948	1824	1888	1921	1938	1928	1856	1843	2004
Standard deviation (K)	49	44	36	26	31	27	38	32	31	36	37
Solid density (${\text {g cm}}^{-3}$)	3.29	4.26	4.68	4.78	4.67	5.14	-	5.18	5.12	5.21	5.17
Liquid density (${\text {g cm}}^{-3}$)	–	–	3.54	3.5	–	3.63	–	–	3.75	3.69	3.69

### Densities (Table 2)

Table 2 reports the solid and liquid density estimates obtained for the different compositions. A slightly closed porosity is observable at the bottom of the droplets, but has not been taken into account in those estimates. ${\text {La}}_2 {\text {S}}_3$ has a measured solid density of $5.2 \, {\text {g cm}}^{-3}$, lowered to $3.7 \, {\text {g cm}}^{-3}$ around 2050 K in its liquid state. The solid density of BaS post experiment is $3.3 \, {\text {g cm}}^{-3}$, however this value is highly affected by the presence of porosity. The liquid density is not reported here due to the decomposition of BaS in temperature.

From 50 to 100 mol% ${\text {La}}_2 {\text {S}}_3$, the solid densities are approximately independent of composition at around $5.2 \, {\text {g cm}}^{-3}$ while the liquid density is around $3.7 \, {\text {g cm}}^{-3}$. From 10 to 40 mol% ${\text {La}}_2 {\text {S}}_3$, the solid density varies from 4.3 to $4.8 \, {\text {g cm}}^{-3}$, and is around $4.7 \, {\text {g cm}}^{-3}$ at 40 mol% ${\text {La}}_2 {\text {S}}_3$. The liquid density in the range 10 to 40 mol% ${\text {La}}_2 {\text {S}}_3$ fluctuates around $3.5 \, {\text {g cm}}^{-3}$. However values obtained for solid and liquid densities in the case of BaS-rich samples are impacted by the difficulty to determine the frontier between the solid and the liquid.

**Figure 7:**
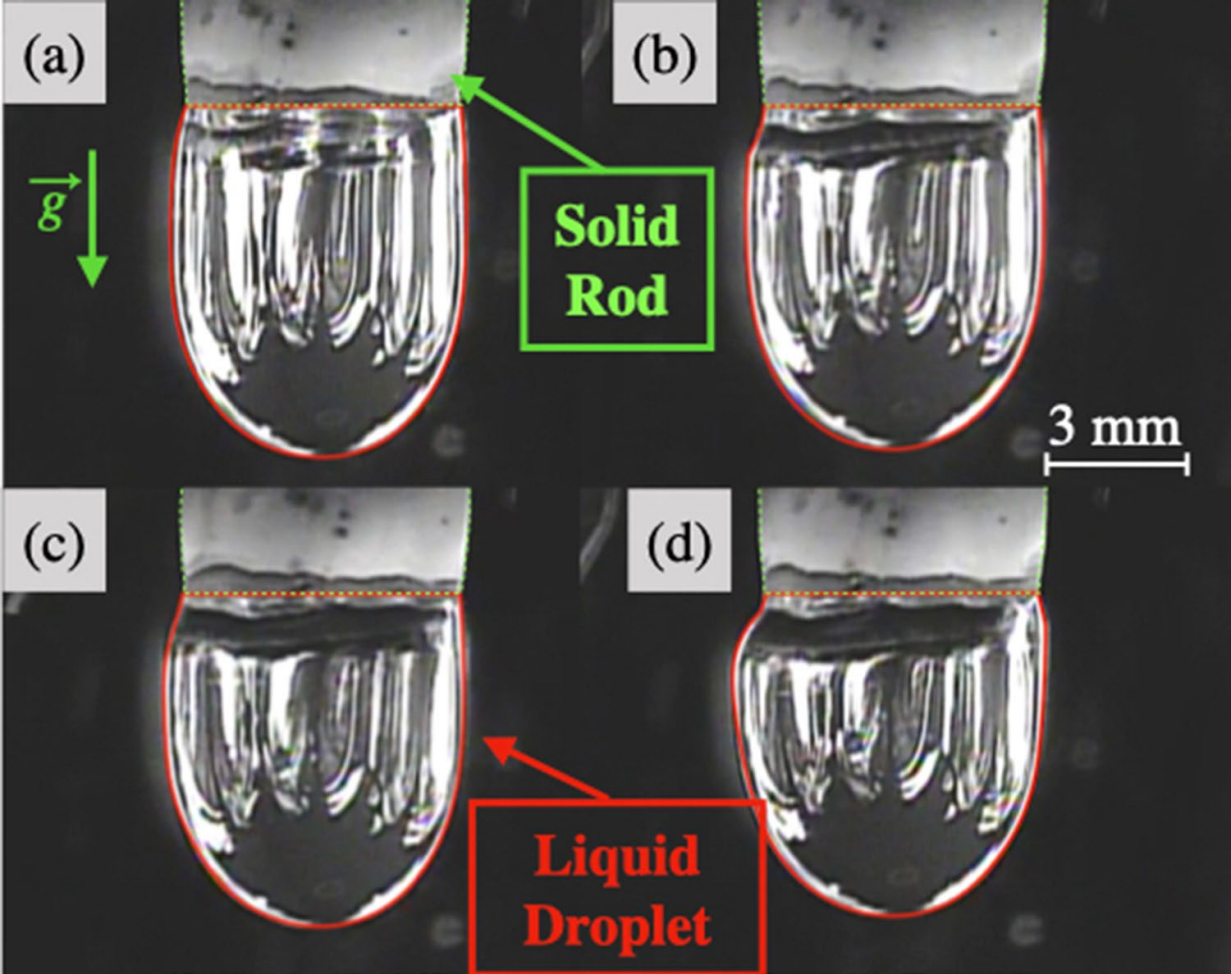
Pictures of ${\text {La}}_2 {\text {S}}_3$ droplet at (**a**) 2063 K, (**b**) 2051 K, (**c**) 2032 K, (**d**) 2017 K allowing the visualization of the variation of volume in temperature above the melting point and therefore the determination of the volumetric isobaric thermal expansion coefficient. (Camera Watec equipped with a UV filter).

### Thermodynamic approach of the volumetric isobaric thermal expansion coefficient $\alpha _p$ for ${\text {La}}_2 {\text {S}}_3$

Figure 7 shows ${\text {La}}_2 {\text {S}}_3$ sample at four different temperatures above the melting point. The temperatures considered are only reaching 60 K above the melting point. In order to maintain a quantity of matter constant at different temperatures, the origin of the considered droplet is set up at the solid/liquid interface of the highest measured temperature. This involves the consideration of a small solidified part for the lower temperature as observable in Fig. 7b–d. The results indicates a volumetric isobaric thermal coefficient of $3.4\text {x}10^{-3} \, {\text {K}}^{-1}$ with a standard deviation of $3\text {x}10^{-4} \, {\text {K}}^{-1}$.

## Discussion

To the authors’ best knowledge,for the first time this experimental design is used in the study of liquid state properties of high temperature sulfide compounds. Herein is demonstrated the range of possibilities of the thermal imaging furnace in the study of liquid state properties of high temperature compounds hardly attainable with other apparatus.

### BaS and ${\text {La}}_2 {\text {S}}_3$

The melting point of BaS reported here is in good agreement with the range of values reported in the literature: 2508, 2480, 2475, 2470 and 2430 K (respectively^6–10^). The small shift found in the literature can be due to the uncertainty of measurements, the high instability of ${\text {BaS}}_{(l)}$ and the possible presence of impurities. ${\text {Ba}}_{(l)}$ and ${\text {S}}_{2(l)}$ vaporize at 2170 K and 713 K respectively; ${\text {BaS}}_{(s)}$ therefore decomposes directly into ${\text {Ba}}_{(g)}$ and ${\text {S}}_{2(g)}$ above 2454 K. The absence of liquid ${\text {Ba}}_{(l)}$ into the remaining sample in the present study confirms this.

Regarding ${\text {La}}_2 {\text {S}}_3$, the observed melting point here at 2004 K is a few hundred Kelvins lower than the values indicated in the literature^10,33,34^. Notwithstanding, Bolgar *et al.* reported in 1986 the change of enthalpy with temperature for ${\text {La}}_2 {\text {S}}_3$^47^. A change of slope is noticeable at 2000 K, matching with the melting point reported herein. Unfortunately Bolgar *et al.* did not address melting or boiling points. The few volume of gas generated and the marginal mass loss indicate a boiling point higher than the melting point and a low partial pressure around 2000 K. The small volume of gas generated herein could be the consequence of the unknown saturation vapor pressure or the presence of impurities. ${\text {La}}_2 {\text {S}}_3$ demonstrates a high stability in its liquid phase not described yet in the literature. From the conclusions presented by Flahaut *et al.*^12^ regarding the strong similarities of ceric sulfides, it can be supposed that all ceric (+3) sulfides exhibit high stability in their liquid state.

### BaS-rich and ${\text {La}}_2 {\text {S}}_3$-rich liquid behavior

The addition of BaS into ${\text {La}}_2 {\text {S}}_3$, from 10 to 40 mol% leads to remarkably stable ${\text {La}}_2 {\text {S}}_3$-rich melts, similar to pure ${\text {La}}_2 {\text {S}}_3$. The thin frontier between the solid and liquid, and the fast homogenization of the temperature inside the droplet indicate good thermal conduction. Added to a mass loss of a few percent, it can be concluded that ${\text {La}}_2 {\text {S}}_3$ could be used as a liquid host to stabilize less stable compounds such as BaS.

The addition of few percent of ${\text {La}}_2 {\text {S}}_3$ into BaS leads to an important decrease of the liquidus. Several studies^3,6,7^ make the case for a similar phenomenon for other additions to BaS. The addition of ${\text {La}}_2 {\text {S}}_3$ favorably stabilizes BaS with temperature, however the presence of two immiscible liquids is not observed as in the case of BaS-${\text {Cu}}_2$S^6^. The liquid phase has a longer lifetime than pure BaS, nevertheless the formation of a neck at the solid-liquid frontier is visible over time.

A strong thermal gradient is observed in the case of BaS-rich samples. Figure 4 demonstrates different regions as a function of the distance from the hot zone. A important sintering process is also observed at the top, likely a consequence of the thermal gradient. The neck formation over time could be directly linked to the low thermal diffusion of BaS-rich samples 4[c-d]. Gravity might also play a key role in the non-homogeneous melting. Complementary studies are required to undertake the complex liquid behavior of BaS-rich samples.

### Porosity

A macropore is observed for ${\text {La}}_2 {\text {S}}_3$-rich samples and regardless of the temperature. During the first heat up, the chemical formation of a sulfuric gaseous compound lead to the formation of microbubbles in addition to the micropores. These microbubbles can reach the surface and leave the system or agglomerate in the center of the molten droplet. The surface tension being too high, the trapped bubble cannot escape and result in a macropore.

For BaS-rich samples, a macropore is also observed for short term experiments and has the same origin as for ${\text {La}}_2 {\text {S}}_3$-rich samples. During long term experiments such as for ${\text {(BaS)}}_{0.75}$-$({\text {La}}_2 {\text {S}}_3)_{0.25}$ samples, the decrease of the liquid volume by the neck formation potentially leads to an expulsion of the gaseous phase. The remaining porosity in ${\text {(BaS)}}_{0.75}$-$({\text {La}}_2 {\text {S}}_3)_{0.25}$ experiments would certainly leave and thus create a porosity free sample if the experiments were conducted for a longer time. The elimination of the porosity in BaS-rich sample seems to be a slow process, equivalent to the neck formation.

**Figure 8:**
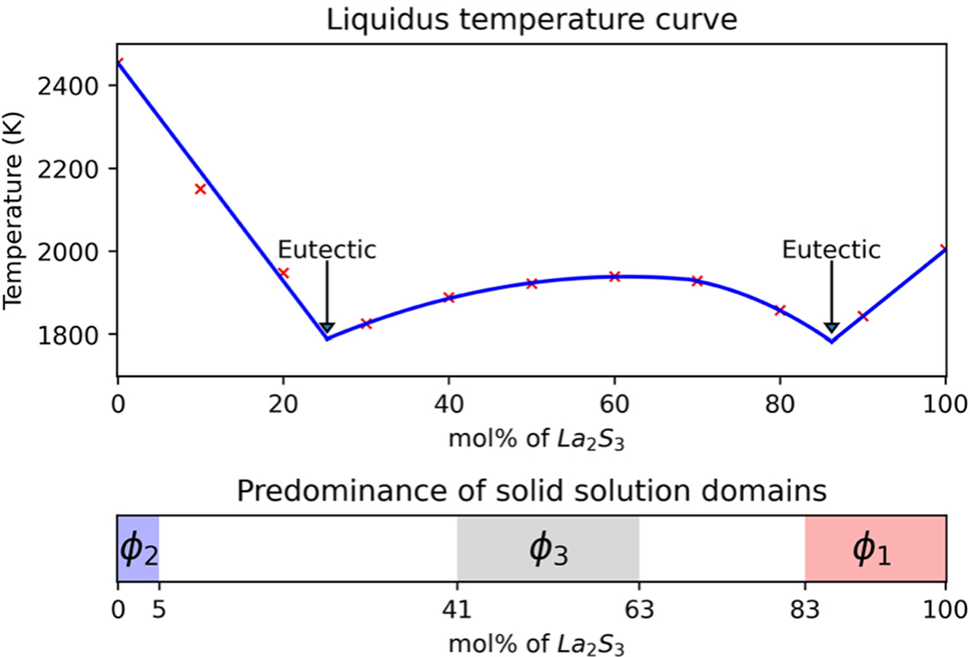
Liquidus temperature for the BaS-${\text {La}}_2 {\text {S}}_3$ pseudo binary system and definition of the potential domains of phase stability and proposed eutectic points (25 and 86 mol% of ${\text {La}}_2 {\text {S}}_3$).

### BaS and ${\text {La}}_2 {\text {S}}_3$ phase diagram

Ideally, experiments should be conducted with slow temperature variations in order to observe only thermodynamic stable phases. In addition, X-Rays Diffraction (XRD) and WDS analysis would bring the complementary information to conclude about the solid state nature, the sulfur concentration and the ratio of metallic elements in the different solid solutions. From the study of the ratio of metallic elements and the literature, assumptions have been made regarding the stoichiometric number of elemental sulfur. The relatively fast cooling processes seen herein may lead to metastable phases, possibly explaining the presence of $\phi _1$, $\phi _2$ and $\phi _3$ solid solutions with variable compositions within a same sample.

$\phi _1$ could be characteristic of ${\text {La}}_2 {\text {S}}_3$ solid solution with insertion of BaS within a minimum concentration of 83 mol% ${\text {La}}_2 {\text {S}}_3$. Andreev and Khritohin already demonstrated the presence of a solid solution of ${\text {La}}_2 {\text {S}}_3$ containing few percents of MnS and MgS^20,21^. When combined with SrS or EuS however, ${\text {La}}_2 {\text {S}}_3$ may demonstrate a large solubility reaching 50 mol%^22,23^.

$\phi _2$ potentially describes BaS solid-solution with addition of ${\text {La}}_2 {\text {S}}_3$ within a maximum concentration of 5 mol% ${\text {La}}_2 {\text {S}}_3$. Andreev and Khritohin also confirmed the presence of a solid solution of BaS containing few percents of ${\text {Lu}}_2{\text {S}}_3$, ${\text {Pr}}_2{\text {S}}_3$, ${\text {Sm}}_2{\text {S}}_3$, ${\text {Tb}}_2{\text {S}}_3$, ${\text {Y}}_2{\text {S}}_3$ or ${\text {Nd}}_2{\text {S}}_4$: rare earth sesquisulfides with properties similar to ${\text {La}}_2 {\text {S}}_3$^8,24^.

$\phi _3$ is a third solid solution with a composition ranging from 41 to 63 mol% ${\text {La}}_2 {\text {S}}_3$. The median composition of this solid solution is around 50 mol% ${\text {La}}_2 {\text {S}}_3$, leading to a potential $\phi _3$ solid solution built around ${\text {BaLa}}_2{\text {S}}_4$. However, ${\text {BaLa}}_2{\text {S}}_4$ has not been reported and is not observed here. The phase diagrams of BaS-${\text {Sm}}_2{\text {S}}_3$ and SrS-${\text {Tb}}_2{\text {S}}_3$ also show a third solid solution. But these phases have a shorter stability domain than observed herein and occur where ${\text {MLn}}_2{\text {S}}_4$ (M = Ba, Sr; Ln = Sm, Tb) represents a limit and not the median composition^24,25^.

Figure 8 represents the liquidus temperature over the BaS-${\text {La}}_2 {\text {S}}_3$ pseudo-binary composition range. $\phi _1$, $\phi _2$ and $\phi _3$ solid solutions are represented in their stability domain observed though the experiments. Traces of $\phi _1$ in the BaS-rich samples and those of $\phi _3$ at 90 mol% ${\text {La}}_2 {\text {S}}_3$ are not considered. These traces may be the consequence of a fast cooling process, leading to thermodynamic unstable phases. Two eutectic points are hypothesized at 25 mol% and 86 mol% ${\text {La}}_2 {\text {S}}_3$.

### Solid density

The solid density of ${\text {La}}_2 {\text {S}}_3$, herein $5.2 \, {\text {g cm}}^{-3}$, is higher than the $5.0 \, {\text {g cm}}^{-3}$ reported literature value^17^. An experiment with another density measurement apparatus and a study of the crystal structure by XRD would be able to confirm or deny the result. A possible explanation to this difference could be the generation of a new crystal structure for ${\text {La}}_2 {\text {S}}_3$: the solidification process from its liquid state does not crystallize in the $\gamma$, $\beta$ or $\alpha$-${\text {La}}_2 {\text {S}}_3$ form, but into another unreported crystal structure denser than the ones reported so far. The solid density of BaS post experiment reaches $3.3 \, {\text {g cm}}^{-3}$, a value highly affected by the porosity hence discarded.

Except for ${\text {La}}_2 {\text {S}}_3$ and BaS, the presence of at least two solid solutions involves an apparent density function of the proportion of each solid solution and their respective composition. The solid density along the ${\text {La}}_2 {\text {S}}_3$-rich samples seems constant around $5.2 \, {\text {g cm}}^{-3}$. The values calculated on the BaS-rich samples are impacted by the presence of open and closed porosity. In addition the thermal insulator behavior of BaS-rich samples makes the frontier between the molten part and the rest of the rod difficult to determine.

### Liquid density

No literature discussing the density of rare earth sulfide in temperature has been found. As in the case of metallic sulfide^48^, BaS-${\text {La}}_2 {\text {S}}_3$ density decreases with the increase of temperature. The difference between solid and liquid densities fluctuate between 1 and $1.5 \, {\text {g cm}}^{-3}$, which are typical values reported for metallic sulfides. The small sample size, imperfect radial homogeneity, neck formation and porosity affect the results. A study with larger samples may be beneficial to increase the precision in the liquid density estimation.

### Volumetric isobaric thermal expansion coefficient $\alpha _p$ for ${\text {La}}_2 {\text {S}}_3$

Using two different samples, the isobaric thermal expansion coefficient was estimated around $3.4\text {x}10^{-3} \, {\text {K}}^{-1}$ with a standard deviation of $3\text {x}10^{-4} \, {\text {K}}^{-1}$. The authors did not find any existing literature discussing this coefficient for liquid rare-earth sulfides.

## Conclusion

High temperature liquid sulfide compounds were analyzed using a container-less thermal imaging furnace. Direct visualization of melting and solidification processes allowed to verify the thermal trace obtained from simple thermometry. Liquid properties such as stability, evaporation rate and liquid density were investigated. The melting point of BaS and ${\text {La}}_2 {\text {S}}_3$ were re-evaluated to 2454 K and 2004 K respectively. The high stability of liquid ${\text {La}}_2 {\text {S}}_3$ was observed while the sublimation of BaS was visually confirmed. BaS, unstable in its liquid state, partially stabilized with the addition of ${\text {La}}_2 {\text {S}}_3$. Degassing and neck formation created difficulties in the study of BaS-rich samples. ${\text {La}}_2 {\text {S}}_3$-rich liquids demonstrated for their part a stunning stability.
